# P-1513. *In Vitro* Activity of Ceftaroline against Bacterial Pathogens from Pediatric Patients Collected During the ATLAS Global Surveillance Program in 2018-2022

**DOI:** 10.1093/ofid/ofae631.1682

**Published:** 2025-01-29

**Authors:** Meredith Hackel, Gregory Stone, Daniel F Sahm

**Affiliations:** IHMA, Schaumburg, Illinois; Pfizer, Inc., Groton, Connecticut; IHMA, Schaumburg, Illinois

## Abstract

**Background:**

The parenteral cephem ceftaroline fosamil is approved for treatment of adults and children with complicated skin and soft tissue infections (SSTI) and community-acquired bacterial pneumonia (CABP) caused by susceptible isolates of *Staphylococcus aureus* (methicillin-resistant [MRSA; SSTI] and methicillin-susceptible [MSSA; SSTI and CABP]), β-hemolytic streptococci (*Streptococcus pyogenes* and *Streptococcus agalactiae* [SSTI]); *Streptococcus pneumoniae* (CABP, including concurrent bacteremia), *Haemophilus influenzae* (CABP), and isolates of *Escherichia coli*, *Klebsiella pneumoniae* and *Klebsiella oxytoca* (SSTI and CABP). Several pathogens causing CABP are also frequently associated with bloodstream infections (BSI). This study examined the *in vitro* susceptibility to ceftaroline and comparator agents among pathogens isolated from pediatric patients with SSTI, respiratory tract infections (RTI), and BSI as part of the Antimicrobial Testing Leadership and Surveillance (ATLAS) surveillance program in 2018-2022.

**Figure:**
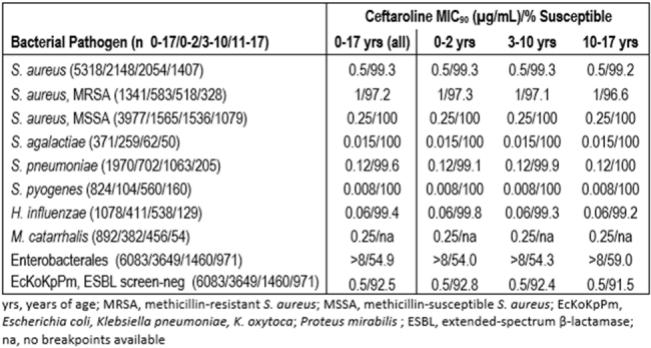

**Methods:**

16,533 non-duplicate, clinically relevant isolates of *S. aureus*, β-hemolytic streptococci, *S. pneumoniae*, *H. influenzae*, *M. catarrhalis* and Enterobacterales were collected from patients aged 0-17 years old with BSI, RTI and SSTI by 285 medical centres in 60 countries. *In vitro* susceptibility to ceftaroline was determined by CLSI broth microdilution and results were interpreted using CLSI 2024 breakpoints where available. Extended spectrum β-lactamase (ESBL) screening was performed using the CLSI M100 method.

**Results:**

The *in vitro* activity of ceftaroline is summarized in the table. 100% of MSSA and β-hemolytic streptococci isolates, >99% of *S. pneumoniae*, >99% of *H. influenzae*, and >92% of ESBL screen-negative *E. coli*, *K. pneumoniae*, *K. oxytoca* and *P. mirabilis* collected from all pediatric patients combined tested as susceptible to ceftaroline, as did >97% of MRSA. *M. catarrhalis* ceftaroline MIC values ranged from ≤0.06 - 8 µg/mL, with an MIC_90_ of 0.25 mg/L. Susceptibility was consistant across each of the age groups.

**Conclusion:**

Ceftaroline demonstrated potent *in vitro* activity against a global collection of clinically relevant pathogens collected from pediatric patients.

**Disclosures:**

**Daniel F. Sahm, PhD**, Pfizer, Inc.: Advisor/Consultant

